# Kidney-Sparing Surgery for Upper Tract Urothelial Carcinoma—Modalities, Outcomes, and Limitations

**DOI:** 10.3390/jcm13216593

**Published:** 2024-11-02

**Authors:** Dennis J. Head, Jay D. Raman

**Affiliations:** Department of Urology, Penn State Health Milton S. Hershey Medical Center, Hershey, PA 17033, USA

**Keywords:** nephroureterectomy, ureterectomy, ablation, chemoablation, recurrence, stricture, preservation

## Abstract

Upper tract urothelial carcinoma (UTUC) accounts for 5–10% of urothelial cancers and is associated with high morbidity and mortality. Increasing incidence of UTUC has been observed since the 1970’s, alongside the evolution of advance imaging techniques, precision biopsy equipment, and risk stratification models. The high morbidity of radical nephroureterectomy (RNU) which is still the gold-standard treatment for high-risk UTUC, has driven the development of kidney-sparing surgery alternatives for low-risk UTUC. Now, several treatment approaches have outcomes comparable to RNU for low-risk UTUC and guidelines are recommending kidney-sparing surgery for favorable low-risk disease. The main categories of kidney-sparing surgery include segmental ureterectomy, endoscopic ablation, chemoablation, and vascular-targeted phototherapy. These treatments are highly nuanced making them difficult to compare, but for most cases of favorable low-grade disease, we recommend endoscopic laser ablation with optional adjuvant intracavitary therapy. Adverse events associated with kidney-sparing surgery include ureteral stricture, bleeding requiring transfusion, and bladder recurrence of UTUC. Limitations of kidney-sparing surgery include appropriate tissue sampling (contributing to under-grading and under-staging), higher rates of ipsilateral recurrence, and potential for grade and stage progression. Collectively, these may subsequently necessitate RNU. Here, we review the technical variations and evidence behind kidney-sparing therapies as well as their practicality in the real world.

## 1. Introduction

Upper tract urothelial carcinoma (UTUC) accounts for 5–10% of urothelial cancers and is associated with high morbidity and mortality [1]. Alongside the evolution of advance imaging techniques, the incidence of UTUC has increased over the past three decades. An associated stage migration to in situ and local UTUC tumors has refined risk stratification models with an appreciation of kidney-sparing treatment modalities [1,2,3]. Historically, nephroureterectomy (RNU) has been the standard treatment for all types of UTUC due to limited alternative treatment modalities [4].

Ubiquitous use of RNU is detrimental to patients’ short-term and long-term health, since it is associated with a 12–18% risk of serious perioperative complications and the potential for some patients to require dialysis due to progression of chronic kidney disease [5,6]. Long operative times averaging approximately 200 min for open and robotic surgeries as well as widely ranging blood loss are also associated limitations of radical nephroureterectomy [7]. Since the average age of onset of UTUC is 73 years of age, many patients may have comorbidities that would put them at higher risk of complications from RNU [8]. For example, an analysis of the American College of Surgeons National Surgical Quality Improvement Program found that of patients who underwent RNU, 5.3% had Clavien–Dindo IV complications, 13.2% had bleeding requiring blood transfusion, 8.3% had readmission, and 3.8% required re-operative surgery [9]. Such adverse outcomes can dramatically impact quality of life in this inherently elderly patient population.

Endoscopy instrumentation has significantly improved over time. The advancement of ureteroscopic technology now allows for improved urinary tract evaluation and tissue biopsy. Moreover, use of selective upper tract cytology collection as well as improved cross-sectional imaging allows better characterization of tumors compared to previous decades. UTUC is now able to be risk-stratified into groups that helps to determine more objectively if a RNU is truly necessary. Several kidney-sparing surgeries have now been developed with the intent of treating low-risk UTUC with decreased morbidity and similar survival outcomes compared with RNU. Here, we will review the roles of organ-sparing treatments and discuss the latest evidence with these treatment approaches.

## 2. Kidney-Sparing Surgery for UTUC

Though RNU is still the gold-standard treatment for high-risk UTUC, kidney-sparing surgery is considered a reasonable treatment option for low risk UTUC [3,10]. One reason is the significantly lower morbidity and similar cancer-specific survival (CSS) when comparing patients treated ureteroscopically or percutaneously to those who underwent RNU for low-grade and noninvasive UTUC [11]. It is worth noting, however, that while there is less morbidity from kidney-sparing surgery, there exists the potential for progression to RNU after recurrence, with salvage rates ranging from 16.7% for low-grade UTUC to 28.6% for high-grade UTUC in a meta-analysis by Seisen et al. [11]. The possibility of recurrence and progression means that patients must be followed with long-term surveillance ureteroscopy and cytology which can be burdensome on patients and the healthcare system [12]. Additionally, patients must accept that embarking on a course of kidney preservation may require pivoting to more invasive surgery if disease parameters indicate progression. Thus, there is a significant investment from the patient, physician, and healthcare system when embarking on this treatment course.

Kidney-sparing surgery (KSS) allows for the preservation of renal function, although evidence is still limited and slightly mixed as to whether this truly changes rates of chronic kidney disease in the postoperative period [11,13,14]. One retrospective analysis of 426 patients by Dudinec et al. found a significant 12.1% increase in the 5-year cumulative incidence of advanced CKD (eGFR < 30 mL/min/1.73 m^2^) in patients treated with RNU compared to KSS [13]. A large meta-analysis comparing segmental ureterectomy (SU) to RNU found higher post-operative GFR for the SU group (weighted mean difference, 11 mL/min; 95% CI 3–19 mL/min; *p* = 0.007) [15]. Preservation of GFR is highly desirable for a few reasons. Firstly, ample data associates CKD with cardiovascular and all-cause mortality [16,17]. Secondly, CKD with associated physiologic changes often necessitates life-long medical therapies such as anti-hypertensive medications and renal replacement therapies which may also have untoward side effects [18,19]. Finally, low-risk patients may benefit from kidney-sparing surgery, so they are not limited in the possible chemotherapy agents for their lifetime [20]. Specifically, for those patients with eventual disease progression, neoadjuvant therapy with cisplatin may be given to patients undergoing RNU if their GFR is predicted to decrease below the threshold for adjuvant chemotherapy [21]. Similarly, adjuvant cisplatin-based therapy is typically avoided in patients with a GFR < 60 mL/min/1.73 m^2^ [21]. Such considerations are not exclusive to UTUC and would also apply to unfortunate scenarios of developing distinct primary malignancies requiring systemic treatments.

Kidney-sparing surgery approaches are less invasive compared to RNU and can be useful for patients who are not able to be optimized for a long and intensive surgery involving multiple abdominal quadrants. There are also imperative indications for attempting kidney-sparing surgery before proceeding to RNU and these include an anatomic or functional solitary kidney, bilateral UTUC, severe chronic kidney disease, and patients with genetic predispositions to contralateral recurrence (Balkan endemic nephropathy or Lynch syndrome) [3,10,22]. Though there are many advantages to kidney-sparing surgery, veering away from the gold-standard RNU treatment requires detailed consideration of risks for each patient.

## 3. Diagnostic Techniques

Evaluation of UTUC includes the results of advanced imaging, fluid cytology, ureteroscopy findings, and biopsy. The AUA recommendation for diagnostic imaging is multiphasic contrast-enhanced computerized tomography urography (CTU), although magnetic resonance urography may be used for patients with iodinated contrast allergy or CKD [10]. A metanalysis of five studies reported the sensitivity and specificity of CT urography to be 96% (95% CI: 88–100%) and 99% (95% CI: 98–99%), respectively, using studies with the inclusion criteria of hematuria [23]. Magnetic resonance urography has a lower sensitivity for UTUC of 69% and a comparable specificity of 97% compared to CTU [24]. MRU is often a more time intensive study with associated costs generally greater than CTU.

If neither CTU nor MR urography can be performed, evaluation may be performed using retrograde pyelography along with non-contrast enhanced computerized tomography (CT) or magnetic resonance imaging (MRI) [10]. Retrograde pyelography has a reported sensitivity of 96% and specificity of 96%, although retrograde pyelography before RNU has been associated with higher intravesical recurrence of UTUC (63% vs. 19%, *p* < 0.001) [24,25]. Renal ultrasonography can also be clinically useful when evaluating UTUC for patients who cannot undergo contrasted CT and MRI studies, but ultrasound cannot evaluate the entirety of the ureter and is therefore not a standalone imaging technique for UTUC evaluation [10,26]. Contrast-enhanced ultrasound (using a contrast agent made of gas-filled microbubbles) is a relatively new imaging modality that is being explored for use in diagnosing UTUC, but there is not robust enough evidence yet to make recommendations for its use [27].

In addition to imaging, fluid and tissue sampling yield important data points used in risk stratification. Fluid cytology should be obtained from voided urine and from renal cytologic barbotage washing, in which the upper tract is rinsed with saline during ureteroscopy and then the wash fluid is collected for analysis [10]. The analysis of cells is reported using the Paris system, which categorizes cells into high-grade UC, low-grade urothelial neoplasm, suspicious for high-grade UC, negative for high-grade UC, atypical urothelial cells, other malignancies, and nondiagnostic [10,28]. Consistent use of standardized nomenclature is critical for ensuring cytology results of urothelial carcinoma.

Voided urine cytology has a reported sensitivity/specificity of 63%/55% for high-grade disease and 53%/37% for low-grade disease, with an overall false negative rate of 43% in one single-center retrospective study of 176 patients [29]. The study also found that positive results were over-graded in 13% and under-graded in 17% of cases. These figures make voided urine cytology less than ideal when trying to determine the presence and grade of disease, but voided urine cytology still gives easily obtainable data points to consider. It has been separately reported that a positive preoperative voided urine cytology is associated with tumor multifocality, higher grade tumor, higher rate of intravesical recurrence, and shorter recurrence-free survival and cancer-free survival [30]. Therefore, patients with a high-grade voided urine cytology may be counselled on the greater risk of intravesical recurrence and the importance of surveillance cystoscopy.

Renal barbotage, also referred to as selective cytology, should be used in addition to voided urine cytology to directly sample fluid closest to the suspected cancer. Renal barbotage should only be performed unilaterally on the side that is being investigated for UTUC and should be performed prior to contrast instillation for retrograde pyelography [10]. A large metanalysis found the pooled sensitivity and specificity of selective cytology to be 53% and 90%, respectively. When stratified by grade, there was a 46% sensitivity for low-grade and a 70% sensitivity for high-grade tumors based on final pathology [31]. Intuitively, selective renal or ureteral cytology from the affected urinary tract likely has superior performance characteristics when compared to a voided cytology with potential dilution from the contralateral renal unit.

Diagnostic ureteroscopy of the side with suspected UTUC should be performed when feasible. Careful endoscopic annotation is strongly recommended. Specifically, notes should be made regarding any bladder lesions, ureteral lesions, and renal pelvis or calyceal lesions, with comments on appearance (papillary, sessile, or flat), focality, dimensions, and obstruction. Biopsy should be performed except in specific circumstances in which a high-grade tumor is highly likely (e.g., high-grade cytology with large enhancing mass) or when the results will not change the management (e.g., patient prefers expectant management) [10].

Biopsy tools that are currently used include cold cup forceps, ureteroscopic baskets, and cryoprobes (cold probes that tissue freezes to when inserted) [32,33]. The goal of biopsy is to obtain the most representative sample of the UTUC tumor. There is, however, no specific data that recommends one type of biopsy tool versus another. Rather, it is important for clinicians to use the biopsy tools that are availed to them at individual institutions. Importantly, given the relatively small sample size of individual specimens, procuring multiple samples will improve the diagnostic yield.

Although biopsy is a direct sample of the target tissue, it is still not representative of the final pathology in many cases which leads to risk of over or under treating patients if it is over-relied upon. Simon et al. conducted a review of 87 UTUC biopsies from patients with subsequent invasion and found that concordance rates of biopsy and RNU final pathology grading were suboptimal, with 11% (10 cases) being reclassified from low grade to high grade. Additionally, biopsy tended to under-stage disease with muscle invasion being upstaged in 30% (26 cases) [34]. These data are somewhat sobering in that it implies patients may be under-graded or under-staged and receive ablation for invasive high-grade disease when looking at the biopsy alone. Therefore, it is important to consider all the available data together when performing risk stratification. Furthermore, limitations of immunohistochemical analysis of UTUC biopsies argues for continued research in molecular profiling of these tumors to capture biological behavior [35].

Of note, patients with Lynch syndrome have a 14-fold higher risk of developing UTUC and a lifetime risk of UTUC up to 20%, but there is no significant difference in outcomes reported [36,37,38]. Currently, Lynch syndrome is not known to predispose patients to high or low risk of invasive disease once it has developed and is not included in the common risk stratification algorithms [10,36]. Still, patients with UTUC should be questioned about personal and familial history of cancer and tested for Lynch Syndrome if appropriate in order to be evaluated for cancer in other organs.

## 4. Risk Stratification

Risk stratification algorithms differ slightly between the European Association of Urology (EAU) and the American Urologic Association (AUA). Prognosis in the EAU is stratified into low-risk and high-risk UTUC [36]. Low risk must include unifocal disease, tumor size < 2 cm, negative cytology for high-grade disease, low-grade ureteroscopic (URS) biopsy, and no invasive aspect on CT [36]. If any of these criteria are not met, it may be considered high-risk UTUC. The EUA considers high-grade cytology, high-grade biopsy, invasion on CT, and high-grade histologic subtypes to be strong criteria for high-risk UTUC, while multifocality, tumor size > 2 cm, and hydronephrosis are considered weak criteria for high-risk stratification of UTUC [36].

The AUA guidelines stratify risk first by low-grade or high-grade ureteroscopic or percutaneous biopsy and then sub-stratify into favorable and unfavorable UTUC [10]. Favorability is based on results of cytology, radiography (invasion, hydronephrosis, or suspicious lymph nodes), URS appearance including focality, and lower tract involvement [10]. Ablative treatments are preferred for favorable low-risk UTUC and may also be offered for unfavorable low-risk UTUC [10]. Favorable high-risk UTUC may rarely be treated with ablation, though RNU is preferred, and adjuvant or neoadjuvant chemotherapy is recommended based on renal function and patient counseling [10]. Unfavorable high-risk UTUC can be palliatively managed with ablative treatments primarily to treat obstruction and maintain renal function [10].

## 5. Techniques of Kidney-Sparing Surgery for UTUC

(1) Segmental ureterectomy is an extirpative treatment alternative to RNU for UTUC and can be employed in low-risk patients and select high-risk patients [10]. Ideal candidates should have a <1 cm unifocal tumor in the distal 1/3 of the ureter, with adequate bladder mobility to create a tension-free anastomosis [10]. Re-implantation is typically done via a psoas hitch or Boari flap technique [10]. Segmental ureterectomy can also be performed for UTUC in the mid-ureter with subsequent ureteroureterostomy using a spatulation technique [10]. An important aspect to segmental ureterectomy is minimizing urine spillage due to the potential of seeding cancer cells being released into the urine during manipulation [10]. Additionally, extensive evaluation of the upper tract for additional lesions and intraoperative pathology should confirm that all cancer has been removed with clean margins [10].

Lymph node dissection for low-risk disease has no statistically significant outcome benefits based on existing meta-analysis studies [10,39,40]. For high-risk disease, lymph nodes should be taken, though templates may vary [3,10]. Interestingly, in studies of RNU for UTUC, patients who are clinically and pathologically negative for nodal metastasis exhibit improved overall survival with higher lymph node yields, regardless of adjuvant chemotherapy use [36,41,42]. Lenis et al. have reported decreasing lymph node yields during RNU as surgeons have transitioned to robotic-assisted laparoscopic approaches compared to the traditional open approach, so this is a potential area to place more importance during robotic-assisted laparoscopic RNU [42].

Segmental ureterectomy in low-risk and select high-risk patients is associated with non-inferior overall survival compared to RNU, but a shorter 5-year recurrence-free survival (OR 0.64; 95% CI 0.43–0.95; *p* = 0.03) [15]. One study specifically compared SU to RNU for middle or distal ureter UTUC less than 3.5 cm and found no differences in bladder recurrence, local recurrence, distant metastasis, cancer-specific survival, or overall survival at the 4-year follow-up [43]. In appropriately selected patients, SU is an effective treatment option though advanced high-risk UTUC should still be treated with RNU [15]. Importantly, patients undergoing SU require continued surveillance of the ipsilateral and contralateral urinary tract generally with imaging first and subsequent endoscopic visualization as needed.

(2) Endoscopic ablation is currently the preferred primary treatment of favorable low-risk UTUC in most patients [10]. This method can be performed via an antegrade approach through percutaneous access or a retrograde approach through ureteroscopic access [44]. The antegrade approach is more invasive due to percutaneous renal access but allows for use larger caliber instrumentation including flexible and rigid nephroscopes and bipolar loop electroscopes. Additionally, easy instillation of adjuvant chemotherapy agents post-operatively can occur when a percutaneous nephrostomy tube is left in place [44]. The direct and large caliber access to the kidney makes the antegrade approach optimal for more extensive low-grade disease where visualization and extensive resection and/or ablation is needed [44]. Furthermore, lower pole tumors with a sharp infundibular pelvic angle may be better suited by direct percutaneous access. The less invasive retrograde approach is ideal for smaller lesions that are easily visualized and are located in parts of the urinary tract readily accessible by ureteroscopy.

One concern that is raised when considering endoscopic ablation is the potential for intraluminal seeding of cancer cells after instrumentation, which can later cause intravesical recurrence of urothelial carcinoma (UC). Evidence for this phenomenon is from observed increases in intravesical recurrence in patients who received a diagnostic ureteroscopy (dURS) prior to RNU, compared to patients who underwent RNU with no dURS [45,46,47]. In a 2022 meta-analysis by Nowak et al., there was significantly worse intravesical recurrence-free survival for patients who received dURS prior to RNU (HR = 1.44, 95% CI: 1.29–1.61, *p* < 0.001) [48]. It is important to note, however, that this study did not find significance for differences in cancer-specific survival, overall survival after RNU, or metastasis-free survival [48].

The theory of intraluminal seeding via instrumentation is also supported by genomic studies of UTUC cells and cells from intravesical UC recurrence, which have found high rates of clonal relatedness between the cell lines [49,50]. Audenet et al. found that in 29 patients with temporally separated UC of the bladder following RNU for UTUC, all bladder UC cell lines shared a clonal origin with the primary UTUC (*p* < 0.005) [49]. Another clonality study by van Doeveren et al. found paired genomics of UTUC and subsequent UC of the bladder in 11 out of 15 patients [50].

A recommended practice to minimize the intravesical recurrence after endoscopic ablation is to instill one dose of post-operative intravesical chemotherapy to kill any viable cells seeded into the bladder, similar to what is recommended after bladder tumor resection [10,51,52]. More than one dose of post-operative chemotherapy does not appear to incrementally provide any additional reduction in IVR [36,53]. One study suggests that using a ureteral access sheath during ureteroscopy reduces rates of intravesical recurrence (HR 0.2; 95% CI 0.1–0.8; *p* = 0.01) [47]. Thus, use of an access sheath may be considered based on limited evidence [47]. Instillation of drugs into the upper urinary tract (in the absence of perforation as determined by pyelography) may also present a dual effect of decreasing local upper tract recurrence while providing bladder chemoprophylaxis.

Outcomes of endoscopic ablation for low-risk tumors are associated with relatively high rates of recurrence (90%) and grade progression (31%); however, 5-year cancer-specific survival is robust (84%) [54]. The outcomes of laser ablation compared to RNU were directly compared in a retrospective analysis by Rouprêt et al. who found insignificant differences in 5-year disease-specific survival rates (84% for RNU; 81% for ureteroscopy; 80% for percutaneous endoscopy) as well as similar 5-year tumor-free survival rates of 71–75% for low-grade UTUC [55,56]. Another study of endoscopic laser ablation reported progression-free survival rates of 75% in patients with low-grade UTUC at a median follow-up of 4.3 years [57]. This study also found that tumor size ≥ 1 cm and multifocality were not associated with increased rates of disease progression [57]. Importantly, second-look endoscopic evaluation is critical to ensure that the ablation eradicated the disease with reports noting that over 50% of patients will have residual carcinoma following the initial procedure [58].

(3) Chemoablation of UTUC using topical application of chemotherapy agents can be used to treat low-grade UTUC [10]. This is primarily recommended when complete endoscopic ablation is not feasible and may also be used as an adjuvant therapy to endoscopic ablation [10]. The limiting factors for chemoablation efficacy in the urinary tract are dwell time of the active agent being limited by washout from urine drainage and dilution of the active agent due to urine production preventing adequate concentrations of the agent [59]. Currently, the only FDA approved form of chemoablation for primary treatment of low-grade UTUC is a reverse-thermal hydrogel (liquid when cool, solid when warm) containing mitomycin C [60,61]. The cooled liquid is instilled to fill the calyces of the affected kidney, solidifies to a gel at body temperature, and releases high concentrations of mitomycin as it dissolves over 4–6 h [60,61,62].

The primary regimen that has been studied involves instillation every week for 6 weeks as an induction regimen with optional additional monthly treatments [60,61]. This has not been validated as the optimal regimen to balance treatment efficacy and associated adverse events [60,61]. The primary adverse event associated with this treatment is ureteric stenosis, presenting in 29% of patients with six or fewer instillations and 66% of patients with more than six instillations as reported in a phase 3 clinical trial [61]. Woldu et al. reported occurrence of significant ureteral stenosis in 23% in a review of 132 patients treated with mitomycin-containing gel [63]. Other treatment-related adverse events include urinary tract infection (32%), hematuria (32%), flank pain (31%), vomiting (20%), renal impairment (20%), and hydronephrosis (18%) [61].

One possible way to minimize the occurrence of ureteral stenosis is to instill the agent via an antegrade approach as demonstrated in a study by Linehan et al. [64]. They found that Clavien grade 3 ureteral stricture was present in 32% of retrograde cases and only 12% of antegrade cases [64]. Another advantage of the antegrade approach is that percutaneous access can be maintained during the entire induction regimen, so the patient can receive instillations in an office, while retrograde instillation must be performed in a procedure room under sedation [64].

Outcome data of chemoablation is still limited, but a phase III clinical trial of mitomycin-containing reverse-thermal gel found a complete tumor response in 59% (95% CI 47–71; *p* < 0.0001) of patients at a median follow-up of 11 months [60]. The 12-month durability of the complete response was 56%, with 8 having recurrence, and 10 unable to be evaluated [61]. Woldu et al. reported that in multi-institutional retrospective data, the rates of being endoscopically clear at first evaluation following treatment were 37% for primary chemoablation, and 69% when the mitomycin gel was used as an adjuvant treatment [63]. They also reported the size of the tumors treated, which revealed a poor response in patients with ≥1 cm of tumor burden [63]. This suggests that current mitomycin-containing gel may be better suited as an adjuvant treatment, rather than a primary chemoablation treatment for low-grade UTUC. The added benefit to primary endoscopic ablation is unknown and requires investigation in a trial. Notably, the current FDA approval for this therapy is for ablative therapy as opposed to adjuvant instillation.

## 6. Future Directions

One treatment in development is vascular-targeted photodynamic therapy (VTP), which has not been FDA approved in the US but is being studied for low-grade UTUC. The treatment involves injecting inactivated padeliporfin systemically, which is then photoactivated in the area of the tumor using an endoscopic laser. Once activated, the padeliporfin causes formation of reactive oxygen species which damage tumor vasculature, stimulate inflammation, and trigger immune responses towards the cancer cells ultimately leading to tumor necrosis [65].

The phase 3 ENLIGHTED trial (NCT04620239) is currently assessing efficacy of this therapy. A limitation of the treatment is that patients have to take light exposure precautions for up to 48 h after padeliporfin is infused to prevent a photosensitivity reaction [66]. The phase I Trial of WST-11 (TOOKAD Soluble) reported adverse events of flank pain (79%) and hematuria (84%) [67]. Efficacy data of VTP using padeliporfin are promising in other solid tumor malignancies such as prostate cancer but they are yet to be published regarding UTUC [68]. It is unknown how most surgeons will utilize this treatment once it is on the market, whether as a primary or adjuvant therapy. Notably, this therapy would focus treatment effect on known visualized sites of disease. Thus, the remainder of the urothelium in other parts of the kidney and upper tract would be less impacted when compared to the chemoablation strategy discussed above.

Another innovation being studied is the use of immunotherapy agents for UTUC treatment. Systemic immunotherapy agents have demonstrated improvements in overall and progression-free survival for many types of cancer, and intravesical BCG immunotherapy has demonstrated efficacy in bladder cancer [69,70]. For UTUC, little research has been carried out on systemic immunotherapy, but a 10-patient feasibility study named PURE-02 found uncertain response or nonresponse in the majority of high-risk patients after three courses of 200 mg pembrolizumab leading up to RNU [71,72]. Currently, immune checkpoint inhibitors are being studied in reverse-thermal gel formulations for treatment of high-grade non-muscle invasive bladder cancer and this could lead to future applications in UTUC [73]. Our understanding of how these drugs interact with UTUC cells is very limited and they seem to be more resistant to immunotherapy than bladder UC cells for a variety of possible reasons [71]. Both systemic and topical immunotherapy agents should be studied with randomized controlled trials in the context of kidney sparing treatment for UTUC.

Targeted chemotherapies for specific UTUC tumor mutations are also being researched. One notable phase 1b trial by Matin et al. explored targeted fibroblast growth factor inhibition, specifically looking at its efficacy in tumors with genetic mutations in fibroblast growth factor receptor 3, which is the most common UTUC mutation [74]. A response was seen in six out of nine patients and three out of five scheduled RNUs were ultimately able to be treated endoscopically [74]. Further research on biomarker-targeted therapy is ongoing and expected to increase kidney-sparing treatment options.

Similarly, detailed molecular profiling of UTUC cells is being explored as a prognostic indicator [75]. In the future, genomic characterization may be able to help indicate when patients may be safely treated with kidney-sparing surgery, and also when patients should receive neoadjuvant chemotherapy prior to RNU [75]. However, more genomic data from UTUC tumors are needed to improve the prognostic accuracy of genomic sequencing.

Finally, as one considers upper tract therapies, novel delivery systems can allow clinicians to use the same drugs but enhance treatment effect. While chemoablation is one such approach, increasing data in the bladder cancer literature highlights the effectiveness of a drug-eluting device (TAR-200) that permits higher concentrations of drug delivery [76]. Similar concepts may be applicable in the upper urinary tract.

## 7. Conclusions

A variety of kidney-sparing surgeries are available for treatment of low-risk UTUC, and innovations are ongoing in this field. Survival outcomes are generally comparable to RNU for low-risk UTUC, but recurrence rates are significantly higher which affects long-term management. Risk stratification remains challenging with the possibility of under-grading and/or under-staging disease, so patients should be on long-term surveillance after kidney-sparing treatments. The adverse events associated with kidney-sparing surgery are drastically different from those of RNU and techniques to minimize morbidity warrant further investigation.
